# Phospho-AXL is widely expressed in glioblastoma and associated with significant shorter overall survival

**DOI:** 10.18632/oncotarget.18468

**Published:** 2017-06-13

**Authors:** Julia Onken, Peter Vajkoczy, Robert Torka, Claudia Hempt, Victor Patsouris, Frank L. Heppner, Josefine Radke

**Affiliations:** ^1^ Department of Neurosurgery, Charité - Universitätsmedizin Berlin, Berlin, Germany; ^2^ Berlin Institute of Health (BIH), Berlin, Germany; ^3^ Institute of Physiological Chemistry, Martin Luther University of Halle-Wittenberg, Halle/Saale, Germany; ^4^ Department of Neuropathology, Charité - Universitätsmedizin Berlin, Berlin, Germany; ^5^ Cluster of Excellence, NeuroCure, Berlin, Germany; ^6^ German Cancer Consortium (DKTK), Heidelberg, Germany, Partner Site Charité Berlin, Berlin, Germany

**Keywords:** glioblastoma multiforme (GBM), receptor tyrosine kinase AXL (AXL), glomeroid tufts, overall survival, phospho-Axl (P-AXL), Pathology Section

## Abstract

Receptor tyrosine kinase AXL (RTK-AXL) is regarded as a suitable target in glioblastoma (GBM) therapy. Since AXL kinase inhibitors are about to get approval for clinical use, patients with a potential benefit from therapy targeting AXL need to be identified. We therefore assessed the expression pattern of Phospho-AXL (P-AXL), the biologically active form of AXL, in 90 patients with newly diagnosed GBM, which was found to be detectable in 67 patients (corresponding to 74%). We identified three main P-AXL expression patterns: i) exclusively in the tumor vasculature (13%), ii) in areas of hypercellularity (35%), or iii) both, in the tumor vasculature and in hypercellular areas of the tumor tissue (52%). Pattern iii) is associated with significant decrease in overall survival (Hazard ratio 2.349, 95% confidence interval 1.069 to 5.162, *p=0.03). Our data suggest that P-AXL may serve as a therapeutic target in the majority of GBM patients.

## INTRODUCTION

Malignant gliomas are the most common and most aggressive brain tumors due to their highly invasive growth pattern, proliferative capacities and heterogeneity [1, 2]. Prognosis remains poor despite multimodal aggressive therapy with chemotherapy, radiation, and surgery [3, 4]. Further research focuses on the identification of new therapeutic targets [5]. As previously described, the receptor tyrosine kinase (RTK) AXL (AXL) displays a new promising target in glioma therapy [6, 7]. AXL plays role in tumor progression and is involved in epithelial to mesenchymal transition (EMT) in different cancer types [8–12]. It has been shown that overexpression of AXL and its ligand Gas6 in glioblastoma (GBM) tissue is associated with reduced time to progression and overall survival time in these patients [13]. Experimental inhibition of the AXL pathway with dominant negative-mutant glioma cells of the AXL receptor (SF126 AXL-DN) results in reduced glioma growth and prolonged survival in the orthotopic tumor model in mice [14]. Further targeted inactivation of AXL with a small molecule inhibitor BMS-777607 leads to a significant decrease of tumor cell growth *in vitro* and *in vivo* due to increased intratumoral apoptosis, impaired proliferation, invasion and neovascularization [7].

In fact, several specific AXL inhibitors have recently entered clinical trials in combination with selective tyrosine kinase inhibitors (Erlotinib: NCT02424617; BPI-9016M: NCT02478866) or chemotherapeutics like cytarabine (NCT02488408). Furthermore, a monoclonal antibody targeting AXL (YW327.6S2) and an AXL decoy receptor (GL2I.T) are in preclinical development [12, 15].

Since AXL kinase inhibitors are about to get approval in clinical phase I and II trails, patients who may have a benefit from anti-AXL therapy need to be identified. We therefore aimed to investigate the expression profile and pattern of the biologically active AXL receptor (P-AXL) in a representative collection of patients with newly diagnosed GBM.

## RESULTS

### Expression pattern of P-AXL

To further extend previous studies, which focused on the expression pattern of AXL in cancer and especially in glioma tissue [13], we studied the expression pattern of the biologically active AXL receptor (P-AXL) in GBM tissue in order to identify subgroups of patients suitable for future anti-AXL therapy.

**Figure 1:**
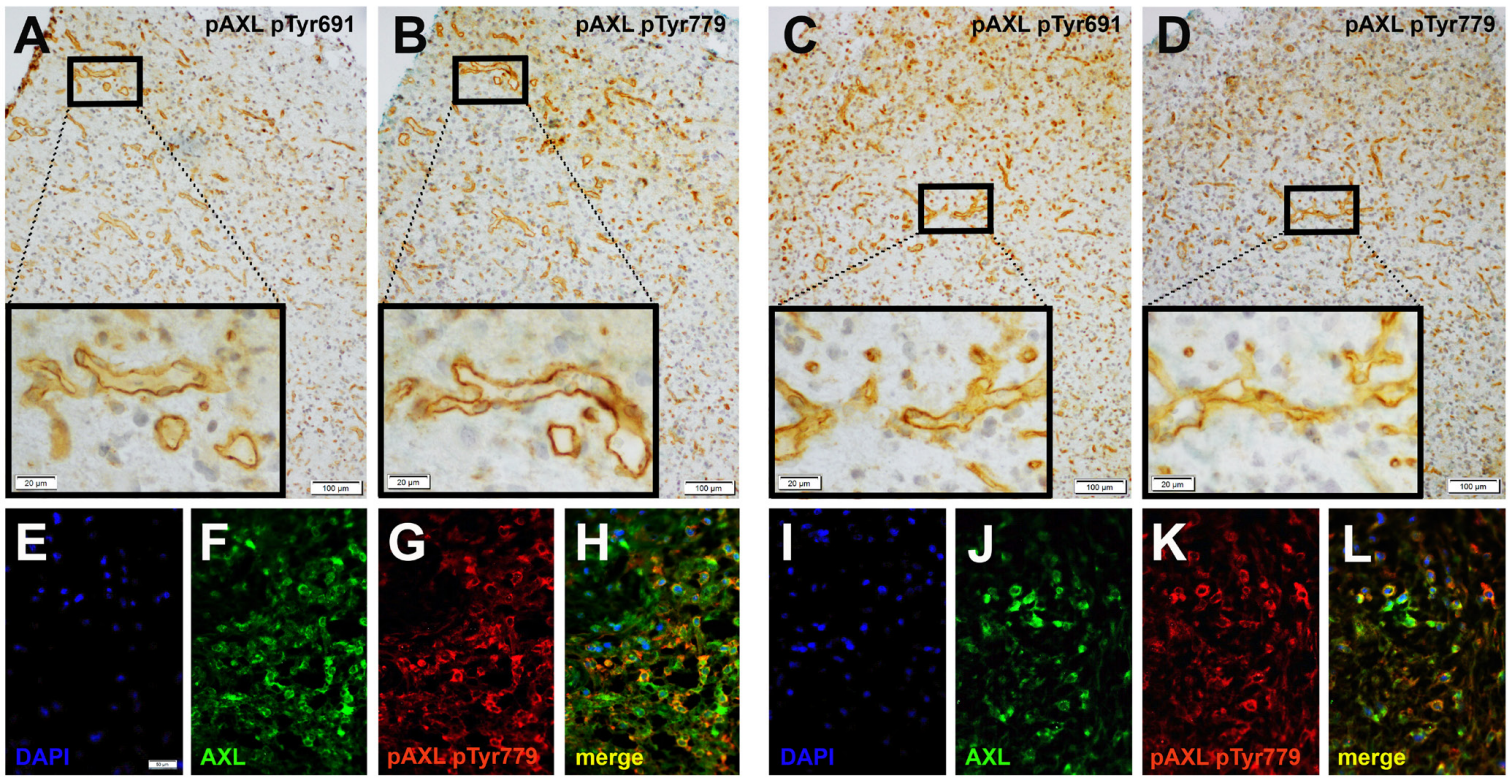
Immunohistochemical evaluation of serial sections of GBM tissue samples revealed the expression of both intracellular AXL phospho-sites - pTyr691 (**A**., **C**.) and pTyr779 (**B**., **D**.) - which were phosphorylated and expressed similarly in the tumor tissue. Immunofluorescent labeling of AXL (**F**., **J**.) and AXL phospho-site pTyr779 (**G**., **K**.) demonstrated colocalization (**H**., **L**.) of both antigens. Counterstaining was performed with 4’, 6-diamidino-2-phenylindoleI (DAPI; **E**., **I**.). (Scale bar: 100 μm **A**.-**D**., 20 μm **A**.-**D**.; inserts), 50 μm **E**.-**L**.)

**Figure 2:**
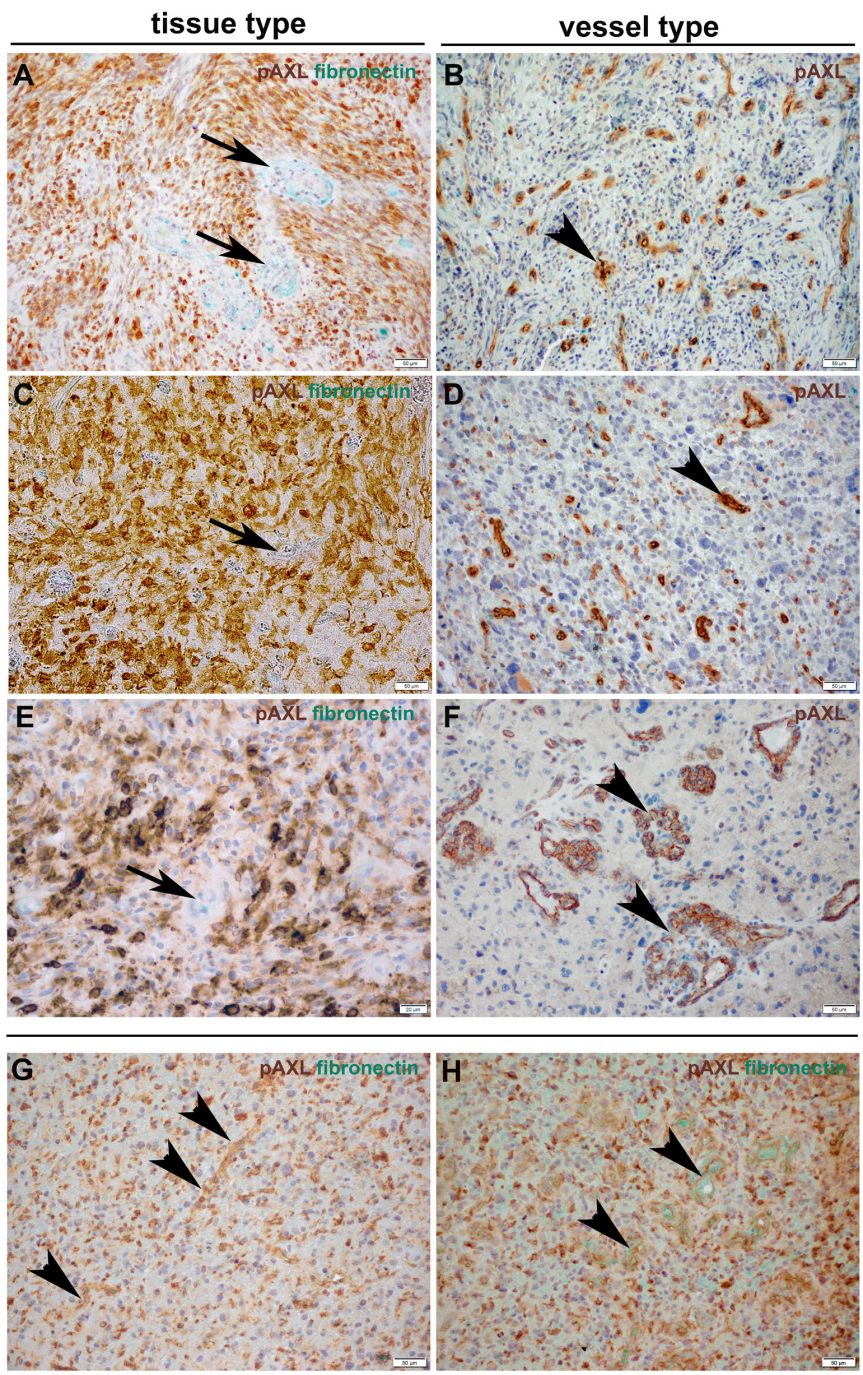
Immunohistochemical double-staining of GBM tissue samples with antibodies against P-AXL (brown) and fibronectin (green) revealed three main P-AXL expression patterns In samples classified as “tissue type”, P-AXL expression was mainly seen in areas of hypercellularity (**A**., **C**., **E**.). Tumor vessels which were highlighted by fibronectin staining were negative for P-AXL (arrows). Samples classified as “vessel type” showed exclusive P-AXL staining within the tumor vasculature (**B**., **D**., **F**.; arrowheads). The third pattern was characterized by P-AXL expression in both, the tumor tissue and vasculature (**G**., **H**.). P-AXL expression was detected in tubular (**B**., **D**., **G**.; arrowheads) and glomeruloid (**F**., **H**.; arrowheads) vascular proliferation. (Scale bar: 50 μm **A**.-**D**., **F**.-**H**.; 20 μm E.).

To confirm the specificity of AXL and P-AXL staining in human formalin-fixed, paraffin-embedded (FFPE) tissue sections (Supplement Figure 1), we used normal brain tissue, known to express very low levels of AXL [16] as negative control (Supplement Figure 1B). Urinary bladder and kidney tissue with known AXL (data not shown) and P-AXL expression served as positive control (Supplement Figure 1A). On serial histological sections, we were able to show that both intracellular AXL phospho-sites pTyr691 and pTyr779 were phosphorylated in GBM tissue (Figures 1A-1D). The expression pattern of P-AXL phosphor-sites pTyr691 and pTyr779 did not significantly differ qualitatively and quantitatively within the tumor tissue (*n* = 10, Figures 1A-1D; inserts). Next, we tested the specificity of the antibody directed against P-AXL *via* the co-staining of AXL and P-AXL (phosphor-sites pTyr691 and pTyr779) using immunofluorescence (Figures 1E-1L). The overall detection rate of P-AXL in GBM tissue was 74% (67 out of 90 patients) in our collective. We identified different P-AXL expression patterns: i) P-AXL expression exclusively in the tumor vasculature (13%, vessel type, Figures 2B, 2D, 2F), ii) P-AXL expression in hypercellular areas of the tumor tissue (35%, tissue type, Figures 2A, 2C, 2E) - here, tumor vessels were immunonegative for P-AXL (Figures 2A, 2C, 2E; arrows) - and iii) P-AXL expression in the tumor vasculature and in hypercellular areas of the tumor tissue (52%, Figures 2G, 2H). Previously, we demonstrated an antiangiogenic effect of AXL inhibition *in vitro* and *in vivo* [7]. There, we showed that P-AXL expression is associated with the characteristic microvascular proliferation in GBMs. In fact, we now can show that P-AXL expression occurs either in glomeruloid tufts (Figure 2F; arrowheads) or in tubular vessels (Figures 2B, 2D; arrowheads) in 65% of patients.

**Figure 3:**
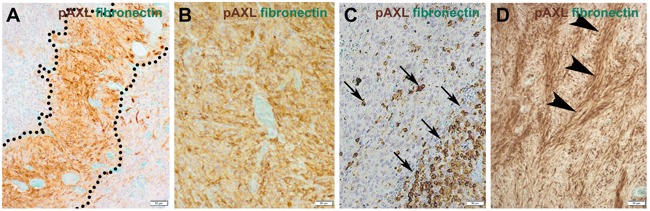
The expression pattern of P-AXL in hypercellular areas was further classified as focal (10-50%; **A**., **C**.) or diffuse (≥ 50%; **B**., **D**.). Focal expression was seen e.g. in pseudopalisades (**A**.; dashed line) or scattered clusters of tumor cells (**C**.; arrows). Other GBM samples demonstrated broad/global P-AXL expression (**B**., **D**.) which was pronounced in areas which often showed a typical herringbone-like pattern (**D**.; arrowheads). (Scale bar: 50 μm).

Prominent microvascular proliferation, cellular atypia, necrosis, brisk mitotic activity and highly cellular areas (pseudopalisades) are characteristic histopathological features of GBM [17]. In fact, 87% of our cases showed P-AXL expression in the hypercellular areas. More specifically, focal P-AXL expression was detected in 66% of the cases (Figures 3A, 3C), while broad P-AXL expression was found in 21% of tumor samples (Figures 3B, 3D). Indeed, P-AXL was frequently expressed in tumor cells located in the perivascular zone as well in the pseudopalisades (Figures 3A; dashed line), which represent highly migratory tumor cells adjacent to hypoxic areas [18]. Apart from that, P-AXL was also strongly expressed in GBM tissue with a herringbone-like pattern (Figure 3D; arrowheads), which was characterized by the presence of elongated tumor cells arranged in fascicles [10]. The herringbone-like phenotype was observed in 26 out of 68 GBM samples, which was accompanied with strong P-AXL expression in 20 (78%) out of 26 samples in these areas (detailed information on the P-AXL expression pattern is provided in Table 1. Gliosarcomas were excluded according to established histologic and immunohistological criteria (GFAP, vimentin and reticulin staining).

**Table 1:** Expression of P-AXL in GBM tissue

P-AXL	yes n (%)	no n (%)	not present
P-AXL staining Hypercellular tumor tissue Focal Diffuse Pseudopalisades Ribbon-like lesions Perivascular Tumor vasculature Glomeruloid tufts and tubular vessels Exclusive vascular	67 (74) 43 (66) 14 (21) 36 (86) 20 (77) 57 (85) 44 (65) 9 (13)	23 (26) 6 (14) 6 (9) 10 (15) 19 (30)	24 (36) 41 (61) 4 (6)

23 out of 90 patients (26%) did not show any P-AXL expression in the tumor parenchyma or tumor vasculature, while we only identified few patients with P-AXL expression exclusively in tubular vessels and glomeruloid tufts, but not in the parenchyma (*n* = 9; 13%).

### Cellular distribution pattern of P-AXL

**Figure 4:**
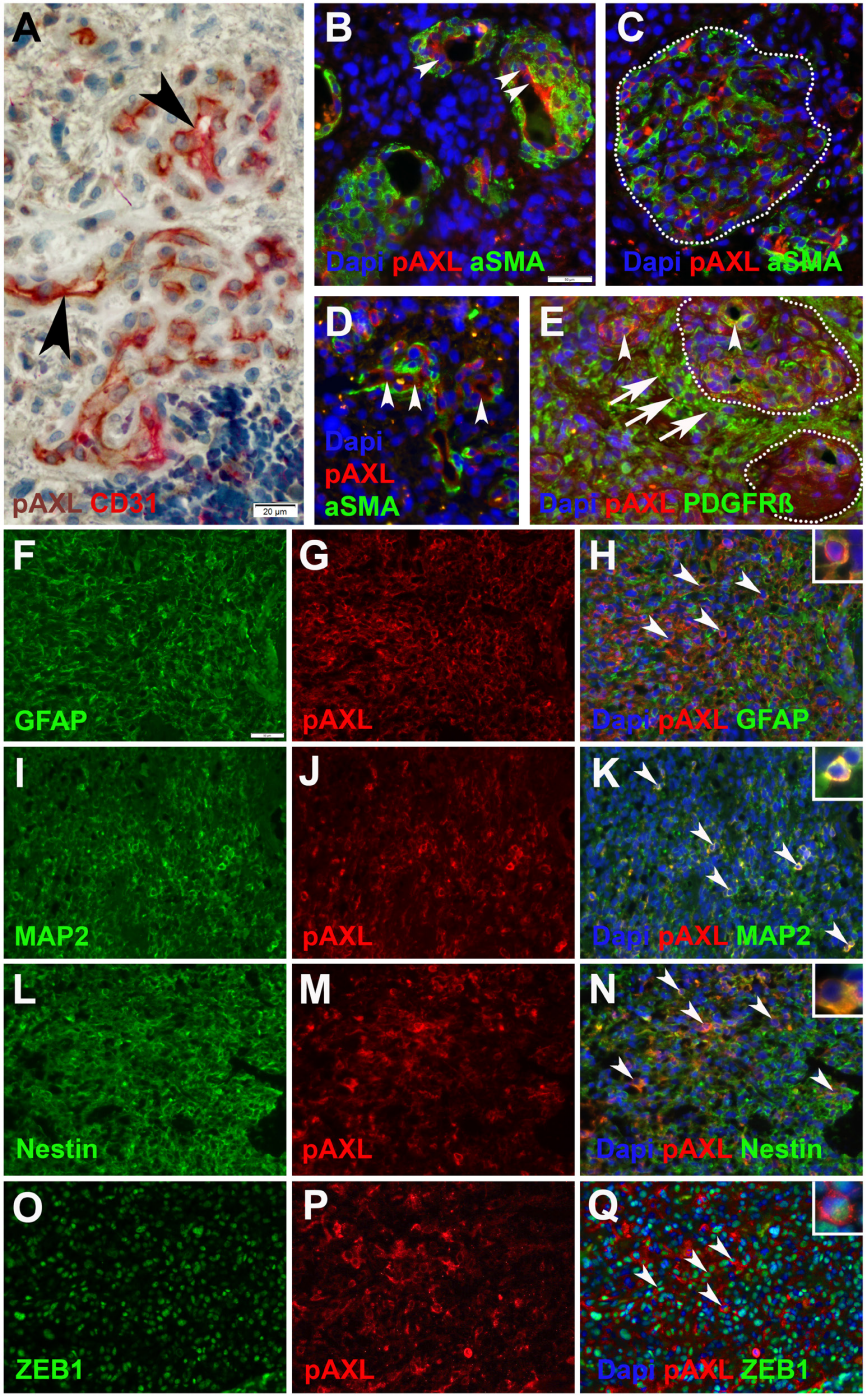
Immunohistochemical and immunofluorescent double-staining of GBM tissue samples revealed strong P-AXL expression in CD31 positive endothelial cells (**A., B**., **D**.; arrowheads), no colocalization with aSMA (**B**., **C**., **D**.), and partial colabeling with PDGFR-ß positive pericytes (**E**.; arrowheads) in microvascular proliferation. Glioma cells adjacent to microvascular proliferation (dashed lines) showed strong immunopositivity for PDGFR-ß (**E**.; arrows). P-AXL was further expressed by neoplastic glioma cells as noted by colabeling with GFAP (**F**.-**H**.; arrowheads), MAP2 (**I**.-**K**.; arrowheads), Nestin (**L**.-**N**.; arrowheads), and ZEB1 **O**.-**Q**.; arrowheads). (Scale bar: 20 μm **A**., 50 μm **B**.-**Q**.).

Double immunohistochemical and immunofluorescent staining were employed to further elucidate the cellular distribution pattern of P-AXL in GBM tissue (Figure 4). P-AXL was strongly expressed in CD31 positive endothelial cells lining the vascular lumens of microvascular proliferation (Figures 4A, 4B, 4D; arrowheads), in glomeruloid tufts (Figures 4C, 4E; dashed line), and partly also in platelet-derived growth factor receptor beta (PDGFR-ß) positive pericytes (Figure 4E; arrowheads). In line with published data, PDGFR-ß staining also highlighted glioma cells [19, 20] (Figure 4E; arrows) adjacent to glomeruloid tufts (Figure 4E; dashed line). In contrast, we detected no clear colocalization of P-AXL and α-smooth muscle actin (aSMA), which was identified primarily in abluminal cells of microvascular proliferation (Figures 4B, 4C, 4D). Whether these aSMA positive cells represent a specialized subgroup of pericytes or cells with smooth-muscle-cell characteristics remains subject of current research and scientific discussion [21, 22]. Next, we found characteristic, cytoplasmic and membrane-accentuated P-AXL staining of glioma cells. In fact, P-AXL was strongly expressed by different cell subpopulation in GBM as indicated by costaining of glial fibrillary acidic protein (GFAP; Figures 4F-4H), microtubule-associated protein 2 (MAP2; Figures 4I-4K), Nestin (Figures 4L-4N), and Zinc finger E-box-binding homeobox 1 (ZEB1, (Figures 4O-4Q).

### Outcome

**Figure 5:**
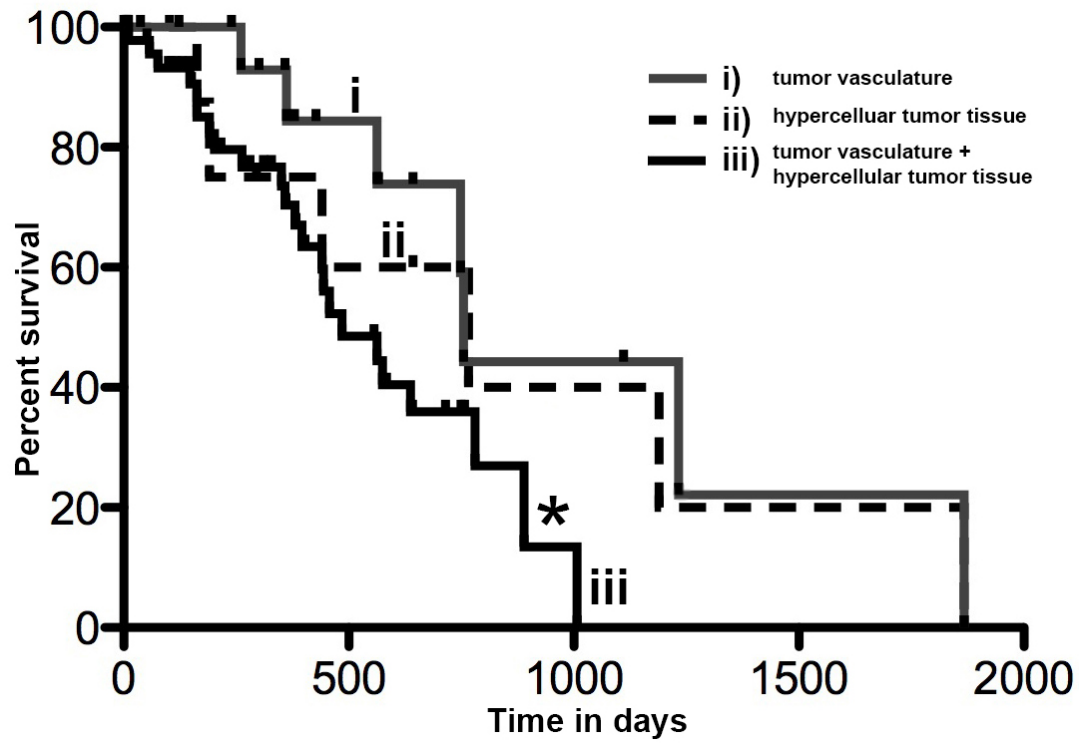
Kaplan Meier curve showing overall survival in days in GBM patients with P-AXL expression i) exclusively in the tumor vasculature, ii) in the hypercellular tumor tissue, or both iii) in tumor vasculature and hypercellular tumor tissue The overall survival of patients with P-AXL expression in vital tumor tissue and tumor vessels (iii) was significantly reduced compared to patients with P-AXL expression in tumor tissue without vascular expression (ii). Log-rank (Mantel-Cox) Test *p = 0.0335, HR 2.349, 95% CI1.069 to 5.162.

We aimed to investigate whether the different tumor expression patterns of P-AXL correlated with the patients’ outcome. We did not find statistical significant differences in progression-free survival (PFS) and overall survival (OS) in the context of global *vs*. focal P-AXL expression (OS: Log rank test, Chi Square, *p* = 0.21; PFS: Log rank test, Chi Square, *p* = 0.12). To further evaluate a correlation of P-AXL staining intensity with the patients’ outcome, we graded all immunopositive P-AXL cases semiquantitatively as follows: 1+ (weak), 2+ (moderate), and 3+ (strong) (Supplement Figures 1G-1I). 32% showed weak (1+), 25% showed moderate (2+), and 43% showed high P-AXL expression (3+). We observed no statistical significant correlation of staining intensity and survival (survival curve comparison of 1+ *vs*. 2+: *p* = 0.3038, CI 0.4874 to 1.090, HR 1.911; survival curve comparison of 1+ *vs*. 3+: *p* = 0.5389, CI 0.5163 to 3.542, HR 1.352; survival curve comparison of 2+ *vs*. 3+: *p* = 0.8279, CI 0.2800 to 2.077, HR 0.8807). P-AXL expression in pseudopalisades or herringbone-like areas was also not associated with tumor progression or survival. Interestingly, our results demonstrated that simultaneous P-AXL expression in the tumor tissue and vessels was associated with significant reduced OS, irrespective of the staining intensity (Log-rank (Mantel-Cox) Test *p = 0.0335, HR 2.349, 95% CI 1.069 to 5.162.). The median survival was 755 days in those GBM patients exhibiting P-AXL expression in hypercellular tumor areas (pattern ii) *versus* 485 days in those with P-AXL expression in the tumor vasculature and hypercellular tumor areas (pattern iii, Figure 5). Univariate Cox regression analysis for outcome relevant parameters such as age, extent of surgery, molecular profile (MGMT and IDH1 (R132) status) and adjuvant therapy, did not show significant differences between the groups (Table 2). Exclusive vascular expression (pattern i) or complete absence of P-AXL did not have an impact on PFS and OS (data not shown).

**Table 2:** Univariate Cox regression analysis (fisher exact test) of P-AXL protein expression in GBM vessels

i) vs. iii)	Odds ratio	95% CI	p
Age 60 vs. >60	1.313	0.4527 to 3.809	ns
Extend of resection GTR vs. PR or Bx	2.045	0.6763 to 6.186	ns
MGMT promoter methylation Hypermethylation vs. no hypermethylation IDH1 (R132) status Wildtype vs. mutant	0.8462 1.361	0.2977 to 2.405 0.1162 to 15.95	ns ns

## DISCUSSION

Our study demonstrates for the first time that the biologically active form of AXL (P-AXL) can be found in 74% of GBM cases, localized either in tumor vessels or in hypercellular tumor areas. Furthermore, we are the first to show that P-AXL is strongly expressed in pseudopalisades, herringbone-like regions and the tumor vasculature of GBM. Also, corresponding expression of P-AXL in hypercellular areas and in the tumor vasculature was shown to be correlated with significant reduced OS. Based on the fact that the majority of GBM cases exhibits a broad expression of P-AXL, our data suggests that P-AXL may represent a suitable target in the treatment of GBM. Our results are also in line with previously published data describing high levels of AXL mRNA (61%) and AXL protein (55%) in GBM patients [13]. The activation of the AXL receptor is known to depend on the binding of its ligand Gas6 to the extracellular domain of the receptor. Gas6 binding leads to dimerization and autophosphorylation of intracellular phosphor-sites of the tyrosine kinase receptor AXL [23]. So far, detailed information providing evidence which phosphor-sites are phosphorylated ligand-dependent or ligand-independent are missing [24, 25]. Here, we demonstrate that apart from the phosphor-site pTyr779, pTyr691 of AXL is also phosphorylated in GBM tissue. The mechanism of action is not known yet, but it can be assumed that ligand-independent activation may also be involved in this process. Gas6 independent activation mechanisms such as hemophilic binding of an extracellular domain of AXL or dimerization and autophosphorylation of AXL or crossphosphorylation of AXL by other receptors like EGFR are equally conceivable [26]. Therefore, it may well be that exclusive inhibition of the AXL/Gas6 axis, as it has been discussed for gliomas and other cancer types [27], might not suffice to prevent AXL activation in GBMs.

Previously, we showed that AXL inhibition by a small molecule inhibitor is leading to a significant decrease of tumor vessel density and tumor vessel size *in vitro* and *in vivo* resulting in decreased tumor volume. The strong and widespread expression of P-AXL in glomeruloid tufts and tubular blood vessels may speak in favor that AXL acts as a driver of tumor angiogenesis. So far, antiangiogenic approaches in glioma therapy did not fulfill expectations [28]. The underlying causes for the development of resistance towards antiangiogenic therapies are not yet fully understood [29]. Interestingly, it has been shown that the activation of the Gas6/AXL pathway promotes both intrinsic and acquired chemotherapeutic resistance [12], including resistance to anti-VEGF therapy [30]. Therefore, a combination of bevacizumab, a monoclonal antibody that specifically recognizes and binds to VEGF, and AXL inhibitors are likely to be a tantalizing anti-GBM approach [27, 30–33]. Moreover, the anti-invasive effect of anti-AXL treatment shown in an orthotopic brain slice invasion assay might provide another argument to combine anti-AXL and anti-VEGF therapy, since invasive growth pattern is another known resistance mechanism in antiangiogenic treatment failure [30].

Recently it has been shown that AXL is a key regulator of the mesenchymal differentiation of GBM stem like cells [34]. The role of AXL in epithelial-to-mesenchymal transition (EMT) has been shown in breast cancer, squamous neck cancer and others malignancies [11, 12, 35, 36]. The predominant expression of P-AXL in herringbone-like areas and pseudopalisades representing highly invasive and migratory tumor cells supports the idea that AXL might also play a role in EMT in GBM [6, 37]. It will thus be of key interest to further evaluate the interplay of EMT mediators, e.g. transcription factors Twist and Snail with AXL expression [12, 34, 38–40].

In conclusion, we herein show that the majority of GBM patients exhibits expression of the biologically active form of the tyrosine receptor AXL in distinct parts of the tumor, indicating that GBMs might be susceptible for anti-AXL therapy. Patients with co-expression of P-AXL in hypercellular areas and in tumor vasculature may benefit the most from such therapy. Combining AXL and VEGF inhibitors might represent a novel approach to circumvent therapy resistance. In fact, since there is good evidence of concomitant activation of multiple RTKs in GBM [41] one can anticipate, that it will be necessary to tailor more “personalized” combinations therapies in order to overcome therapeutic resistance in GBM.

## MATERIALS AND METHODS

### Immunohistochemical and immunofluorescence procedures

Immunofluorescence and immunohistochemical stainings were performed on formalin-fixed, paraffin-embedded (FFPE) tissue sections according to standard procedures. The following primary and secondary antibodies were used: polyclonal rabbit anti-GFAP (1:2000, Dako), monoclonal mouse anti-CD31 (1:100, clone JC70A, Dako), monoclonal mouse anti-MAP2 (1:10000, clone HM-2, Sigma Aldrich), monoclonal mouse anti-α-smooth-muscle actin (aSMA, 1:200, clone 1A4, Dako), monoclonal mouse anti-Nestin (1:200, clone 10C2, Millipore), polyclonal rabbit anti-PDGFR-ß (1:10, Santa Cruz), polyclonal rabbit anti-ZEB1 (1:300, Sigma Aldrich), polyclonal anti-Fibronectin (1:1000, Sigma Aldrich), monoclonal mouse anti-phospho-AXL (pTyr779) (1:50, clone 713610, R&D Systems), polyclonal rabbit anti-phospho-AXL (pTyr691) (1:50, Sigma Aldrich), monoclonal rabbit anti-AXL C89E7 (1:100, Cell Signaling), FITC-conjugated donkey anti-rabbit IgG (1:1000, Dianova, 711-095-152), Alexa Fluor^®^ 488-conjugated goat anti-rabbit IgG (1:1000, Dianova, 111-545-003), Alexa Fluor^®^ 488-conjugated goat anti-mouse IgG (1:1000, Dianova, 115-545-003), Cy3-conjugated goat anti-mouse IgG (1:1000, Dianova, 115-165-003), Cy3-conjugated goat anti-rabbit IgG (1:1000, Dianova, 111-165-003), and Cy3-conjugated donkey anti-mouse IgG (1:1000, Dianova, 115-165-146). The immunofluorescence counterstaining was performed with VECTASHIELD^®^ Mounting Medium containing 4’,6-diamidino-2-phenylindole (DAPI) (Vector Laboratories, Burlingame, CA). Immunohistochemical staining of FFPE tissue sections (4 μm-thick) was performed on a VENTANA Benchmark XT automated staining instrument according to the manufacturer's instructions. Slides were de-paraffinized using EZ prep solution (Ventana Medical Systems, Tucson, AZ) for 30 minutes at 75 °C. Antigen retrieval was accomplished on the automated stainer using CC1 solution (Ventana Medical Systems, Tucson, AZ) for 60 minutes at 95 °C. Briefly, primary antibodies were applied and developed using the iVIEW DAB Detection Kit (Ventana Medical Systems), the Permanent HRP Green Kit (Zytomed Systems), and the ultraView Universal Alkaline Phosphatase Red Detection Kit (Ventana Medical Systems). All slides were counterstained with hematoxylin for 4 minutes. Immunohistochemical results of P-AXL staining intensity were evaluated semiquantitatively by two independent, blinded experts based on the predominant staining intensity (H-score) [42], which was graded as 0 (negative; Supplement Figures 1C, 1D), 1+ (weak; Supplement Figure 1G), 2+ (moderate; Supplement Figure 1H), and 3+ (strong; Supplement Figure 1I). Omission of primary antibodies as control for nonspecific binding of the secondary antibody resulted in absence of any labeling. To validate our immunohistochemical and immunofluorescence stainings we used different positive control tissues fixed and processed in similar manner to the test sections and known to contain the target molecule, e.g. urinary bladder or kidney.

### Microscopy

Images were recorded by using a fluorescent microscope (Zeiss, Obeserver Z1). Following objectives were used: 5x EC PlnN, 5x/0.16 DIC0 (resolution: 2.0 µm), 10x Pln Apo, 10x/0.45 DIC II (resolution: 0.74 µm), 20x Pln Apo, 20x/0.8 DIC II (resolution: 0.42 µm). We use a HAL 100 and detectors for DAPI, GFP and DSRed. Pictures were processed and recorded with Image software Axio Vision Rel. 4.8.

### Histopathological grouping and analysis

GBM tissue sections were analyzed according to their P-AXL expression profile. Analysis was focused on the expression of P-AXL in the histopathological key features of GBM such as microvascular hyperplasia characterized by glomeruloid tufts and tubular vessels and hypercellular regions including pseudopalisades and herringbone-like areas [18]. Hypercellular regions were defined as clusters of highly malignant GFAP-positive tumor cells mostly crowded along the edges of necrotic zones and characterized by prominent eosinophilic cytoplasm, marked nuclear atypia and elevated mitotic activity [43].

The following P-AXL expression patterns were detected: i) P-AXL expression exclusively in the tumor vasculature, ii) P-AXL expression in hypercellular areas of the tumor tissue, and iii) P-AXL expression in the tumor vasculature and in hypercellular areas of the tumor tissue. In pattern i) P-AXL expression was observed in vascular proliferates like glomeruloid tufts and tubular vessels. Within the GFAP-positive, hypercellular regions, the presence of P-AXL in herringbone-like areas or pseudopalisades was documented. The expression pattern of P-AXL within hypercellular regions was further classified as focal (10-50%) or diffuse (≥50%) and was independently assessed by two different, blinded experts.

### Patient data

Clinical data were evaluated under an institutional review board-approved protocol and de-identified for patient confidentiality. We included 90 patients, who have been treated in our institution in the year 2012-2016. GBM diagnosis was confirmed by at least two neuropathologists. Age, tumor localization, Karnofsky performance status (KPS), O(6)-methylguanine-DNA methyltransferase (MGMT) status, isocitrate dehydrogenase 1 (IDH1 (R132)) mutation, extend of resection, adjuvant therapy (irradiation dosage and type/duration of chemotherapy treatment) and outcome data were recorded. Outcome measures were assessed with progression free survival (PFS) and overall survival (OS) in month according to RANO criteria [44]. Prognostic relevant factors like age, MGMT status, IDH status und extend of resection were taken into account for survival analysis. The extend of tumor resection was determined by measuring the contrast-enhancing residual tumor volume in mm^3^ on T1 subtraction MRI imaging using iPlan Net 3.0.0, BrainLAB AG, Feldkirchen, Germany. The extend of resection was defined as gross total resection (GTR) with residual tumor volume less than 1 cm^3^ according to Sanai et al. [45], partial resection (PR) with more than 1 cm^3^ residual tumor volume or biopsy in cases of stereotactic or open tumor biopsy. IDH1 (R132) mutation was assessed with immunohistochemistry (IHC). MGMT promoter methylation status was assessed based on previously published methods with methylation-specific polymerase chain reaction (MSP) and pyrosequencing (PSQ) [46]. Patient characteristics are displayed in Table 3.

**Table 3:** Patient characteristics

n _total_= 67		n	%
Age Extend of resection MGMT status IDH1 (R132) status Irradiation Chemotherapy PFS (month) OS (month)	60 >60 GTR Bx PR n.a. hypermethylation no hypermethylation mutant wildtype n.a. 59-60Gy 34-38Gy n.a. Stupp Nordic glioma no mean range mean range	27 40 41 5 17 4 36 31 5 58 4 44 11 12 49 8 10 10 0-26 16 0-62	40 60 62 7 25 6 54 46 7 87 6 66 16 18 73 12 15

### Statistical analysis

Statistical analysis was performed using GraphPad Prism 5.0c, GraphPad Software Inc, La Jolla, USA. For statistical test we used Student´s *T*-Test and one-way ANOVA combined with Bonferroni´s multiple comparison test. Survival end points matched to date of death or follow-up end points. Patients who were alive at the follow up date were censored for survival analyses. Kaplan-Meier curves were plotted to estimate PFS and OS as a function of P-AXL expression. Univariate Cox regression analysis was used to determine whether prognostic factors like age, extent of surgery, adjuvant therapy, and MGMT or IDH1 (R132) status were differentially distributed in the compared groups. Group differences were assessed with log rank test for trend (Chi-square). Significance level was set at *p* < 0.05.

## SUPPLEMENTARY MATERIALS FIGURES


