# Supplementary material for: Phospho-AXL is widely expressed in glioblastoma and associated with significant shorter overall survival
Source: Oncotarget. 2017 Jun 13;8(31):50403–14. doi: 10.18632/oncotarget.18468 (PMC5584143; doi:10.18632/oncotarget.18468)
Supplement: Supplementary file 1 [file oncotarget-08-50403-s001.pdf]

Phospho-AXL is widely expressed in glioblastoma and associated with significant shorter overall survival

Supplementary Material

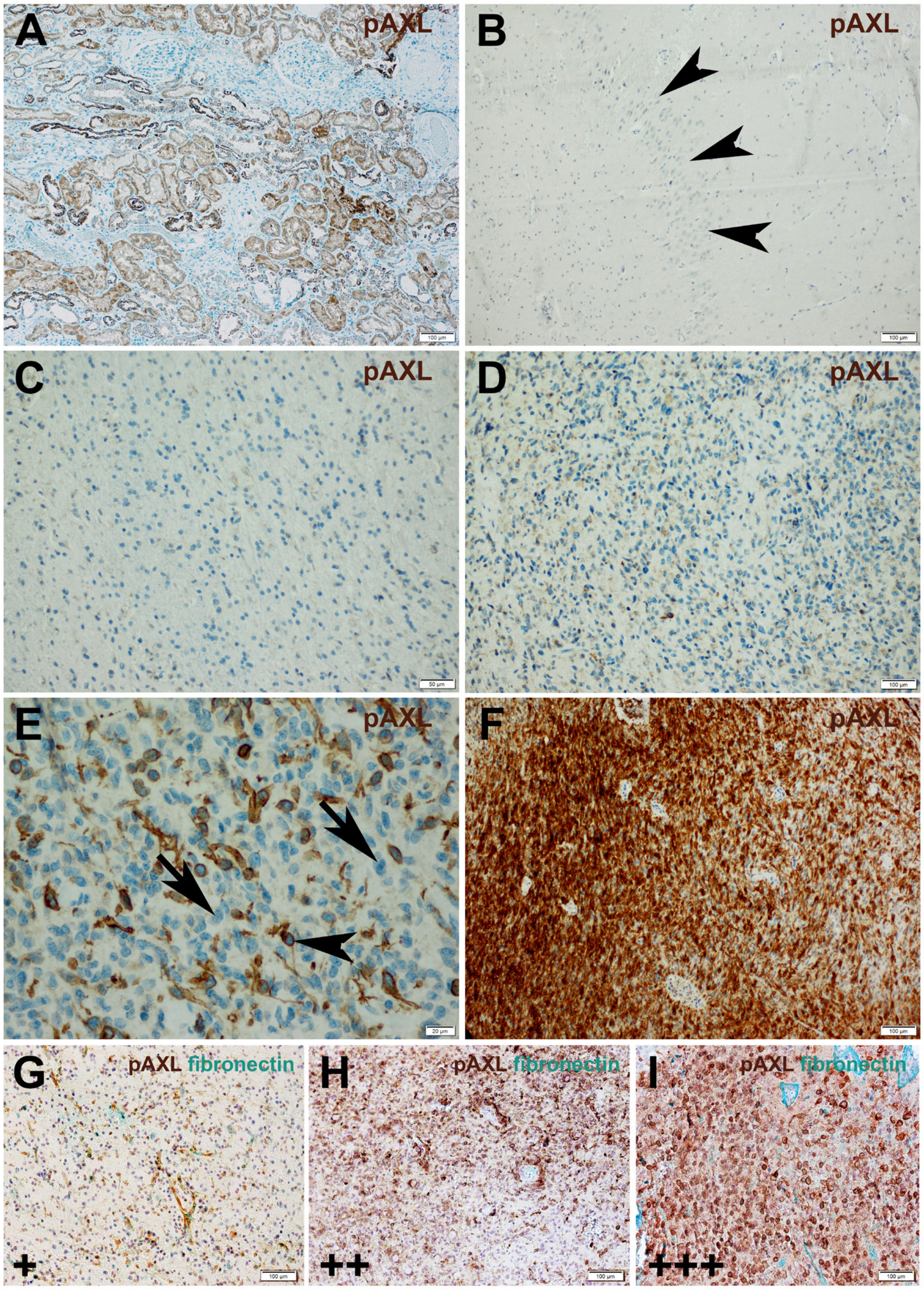

Supplementary Figure 1: To confirm the specificity of P-AXL staining in formalin-fixed, paraffin-embedded (FFPE) tissue sections, human kidney served as positive control (A). Normal brain tissue was used as negative control (**B**; *arrowheads:* dentate gyrus). **C** and **D** illustrate two GBM cases with weak background staining that were considered immunonegative (0) for P-AXL.. Focal P-AXL expression was detected in **E** with clearly positive (arrowhead) and negative (arrows) tumor cells. **F**: Example of a GBM case with global P-AXL expression. Positive staining intensity of P-AXL was graded semiquantitatively as 1+ (weak, *G*), 2+ (moderate, *H*), and 3+ (strong, *I*). (Scale bar: 100 µm (*A, B, D, F, G-I*); 50 µm (*C*); 20 µm (**E**))
